# Polishing of Silicon Nitride Ceramic Balls by Clustered Magnetorheological Finish

**DOI:** 10.3390/mi11030304

**Published:** 2020-03-14

**Authors:** Xiao-lan Xiao, Guang-xian Li, Hai-juan Mei, Qiu-sheng Yan, Hua-tay Lin, Feng-lin Zhang

**Affiliations:** 1 School of Mechanical and Electrical Engineering, Guangdong University of Technology, Guangzhou 510006, China; xxlan@gdut.edu.cn (X.-l.X.); huataylin@comcast.net (H.-t.L.); zhangfl@gdut.edu.cn (F.-l.Z.); 2 School of Engineering, RMIT University, Melbourne, VIC 3000, Australia; guangxian.li@rmit.edu.au; 3 School of Electronic Information and Electrical Engineering, Huizhou University, Huizhou 516007, China; haijuanmei@hzu.edu.cn

**Keywords:** silicon nitride (Si_3_N_4_) balls, clustered magnetorheological finish (CMRF), surface finish, polishing experiments, kinematic modelling

## Abstract

In this study, a novel finishing method, entitled clustered magnetorheological finish (CMRF), was proposed to improve surface finish of the silicon nitride (${\mathrm{Si}}_{3}N_{4}$) balls with ultra fine precision. The effects of different polishing parameters including rotation speeds, eccentricities and the machining gaps on surface finish of ${\mathrm{Si}}_{3}N_{4}$ balls were investigated by analyzing the roughness, sphericity and the micro morphology of the machined surface. The experimental results showed that the polishing parameters significantly influenced the surface finish. The best surface finish was obtained by using the polishing parameters: the machining gap of 0.8 mm, the eccentricity of 10 mm and the rotation ratio of 3/4. To further investigate the influence of the polishing parameters on the surface finish, an analytical model was also developed to analyze the kinematics of the ceramic ball during CMRF process. The resulting surface finish, as a function of different polishing parameters employed, was evaluated by analyzing the visualized finishing trace and the distribution of the contact points. The simulative results showed that the distribution and trace of the contact points changed with different polishing parameters, which was in accordance with the results of experiments.

## 1. Introduction

Silicon nitride (${\mathrm{Si}}_{3}N_{4}$) ceramics are widely used in numerous industrial sectors due to their outstanding physical and mechanical properties [1]. Silicon nitride ceramic balls are the first choice for high-performance ball-bearing materials due to their high hardness, low density, low thermal expansion coefficient, high-temperature resistance, non-magnetic nature, corrosion resistance and self-lubricating property [2]. At present, the common practice of the finishing processes of ${\mathrm{Si}}_{3}N_{4}$ ceramic balls can be achieved through the mechanical action of abrasive grains. For example, V-groove finishing is a typical method employed for the surface finish of spherical parts, which is still widely used in industry currently. However, due to the low surface energy of ceramic balls and poor adhesion at the workpiece/abrasive plate interface [3], the final polishing accuracy and surface condition of ceramic balls are difficult to control, especially for spherical errors [4]. Furthermore, the inevitable defects present on the polished surface significantly limited the efficiency of the V-groove finishing approach. Defects, including scratches, pits, snowflakes, and micro-cracks are often found on polished surfaces due to the continuous abrasion between the abrasive plates and the workpiece surface [5]. Besides the surface defects, the normal and shear stresses present on the abrasive plate could cause the propagation of inner cracks during the finishing process of spherical parts made of ceramic materials [6]. Moreover, adhesion of the debris and chemical diffusion between the workpiece and abrasive plates were observed, which could also cause deterioration of geometric precision and mechanical properties of machined surfaces [7]. 

Some attempts have been performed to improve the efficiency and surface finish of spherical parts. For example, Feng et al. [8] proposed a double-plane polishing method to polish ceramic balls, which aimed to improve the surface finish by dual-plane polishing technology, compared with conventional finishing method using cast iron plates with V-grooves. The material was removed uniformly by making the balls’ spinning and rolling constant between the abrasive plates, and the results showed that the average surface roughness value was achieved with better results, the surface roughness Ra was reduced from 20 nm to 4 nm and the sphericity was maintained within 0.25 μm after 20-h polishing. However, the improvement of spherical error was not promising. Zhao et al. [9] proposed a variable-radius V-groove polishing method by changing the spin angle of the abrasive plate. By adopting this method, the G16 level steel balls were polished to G5 level after 10 revolutions of the lower plate, and the machining time reported was only two minutes. However, the application of the variable-radius V-groove method on the polishing of balls consisting of ceramics was not further investigated. Feng et al. [10] polished the ceramic balls with the spiral V-groove plates aiming to improve the sphericity of the surface finish. In the polishing process, the movement characteristics of the balls changed with the curvature radius of the groove, which increased the uniformity of the polishing on the ball surface. The best surface finish in G3 level was achieved with a roundness of 0.05 µm, surface roughness of 4.5 nm and spherical error of 0.11 µm, but the stability of the polishing process and practicality of this polishing method in industry were still questionable, because no further research was conducted on the surface finish of balls with different sizes.

With the development of polishing techniques, flexible nano-finishing processes have been applied in polishing various materials such as metals, ceramics and optical glasses. These polishing methods, including magnetic float polishing (MFP), magnetic abrasive finishing (MAF) and magnetic compound fluid slurry finishing (MCF), are capable of achieving surface roughnesses in the range of 10–100 nm on metals with flexible finishing medium [11,12,13]. By using soft finishing tools including chemical liquids, electric or magnetic fields, ceramic balls with a super smooth and precision surface finish could be obtained. Umehara et al. [14] reported a new method based on the magnetic float polish (MFP), which resulted in the polished ceramic balls with no surface damages and an ultra-fine surface roughness of less than 4 nm. However, the high cost of magnetic fluid along with the complicated equipment setup limit its potential industrial application. The magnetorheological finish (MRF) approach is known as a novel surface processing method combining electromagnetics, fluid dynamics, analytical chemistry, and processing technology [15]. Compared to traditional polish methods, MRF has the characteristics of low loss, high efficiency, and the processing effect of nanometer precision. Pan et al. [16] proposed a cluster magnetorheological finishing (CMRF) method for the polishing of strontium-titanate ceramic surfaces. The material removal rate was 0.154 µm/min and the final surface roughness was around 8 nm, which demonstrated a good processing efficiency and high quality on the surface finish. Liang et al. [17] also applied CMRF for the polishing of SiC ceramic plane surfaces. Results showed that the final surface roughness was about 0.6 nm when the magnetorheological slurry consisting of carbonyl iron powders and $H_{2}O_{2}$ was applied. Zhao et al. processed the quartz wafer using MRF in order to remove the defects including facial cracks and dimples. The results showed that the surface roughness was reduced from 2.46 nm to 0.653 nm, and the root mean squre (RMS) was reduced from 35.6 nm to 5.06 nm [18]. However, the CMRF method applied in the aforementioned three studies was only performed for the polishing of flat surfaces, further investigation on other types of surface (e.g., free surfaces) and workpiece materials were not conducted.

It is therefore suggested that CMRF is a promising method for finishing ceramic materials according to those aforementioned studies. However, the application of CMRF on the finish of spherical parts made of ceramics has not been reported yet. In this study, the novel application of the CMRF technique for polishing ${\mathrm{Si}}_{3}N_{4}$ ceramic balls were investigated both experimentally and theoretically. A series of polishing experiments with different parameters were conducted on a customized computer numerically controlled (CNC) CMRF machine, and the roughness, sphericity and microstructure morphology were systematically analyzed to evaluate the influence of three main parameters: rotating speeds and eccentricities of the abrasive plates and machining gaps on the surface finish of the ${\mathrm{Si}}_{3}N_{4}\;$ceramic balls. To further explore the mechanism how the polishing parameters affect the surface finish, the kinematics of the CMRF process during the polish was theoretically modelled and dynamically simulated. The visualized finishing trace and the distribution of the contact points were analyzed to explore the influence of different parameters applied on the CMRF processes.

## 2. Experimental Procedures

### 2.1. The Principle of CMRF

**Figure 1:**
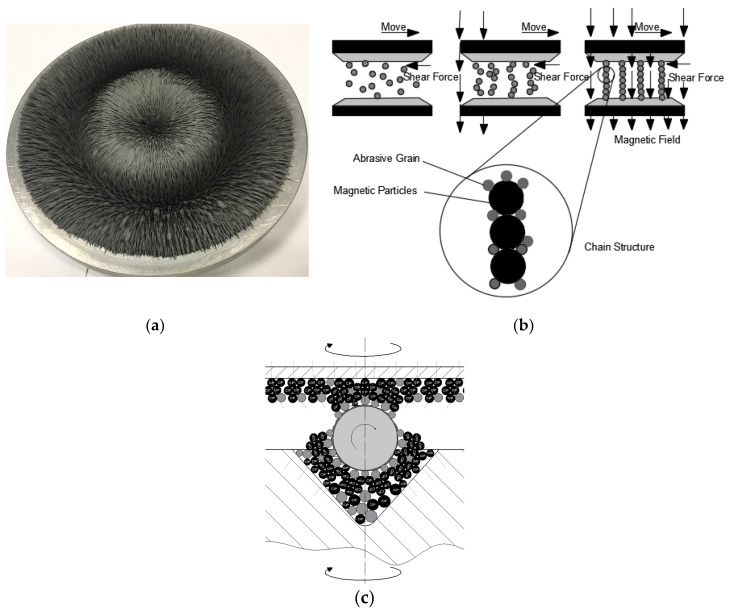
(**a**) the magnetorheological pad (**b**) The formation of the magnet chains (**c**) polishing mechanism of clustered magnetorheological finish (CMRF).

In this study, the CMRF method was applied to improve the surface finish in the polishing of ${\mathrm{Si}}_{3}N_{4}$ ceramic balls. The principle of the material removal process of CMRF is presented in Figure 1. During the polishing process, the surface materials under contact are removed by the abrasion between spherical surface and magnetorheological pads. With the supplement of electricity, the slurry, which consists of iron particles and abrasive grains, is shaped into a series of magnetic chains due to the cluster magnetorheological effect [19], which in turn forms the magnetorheological finishing pads on the surfaces of the upper and lower plates (Figure 1a). 

By driving the rotating shafts of upper and lower finishing plates, the ceramic balls rotated and were polished under the shear force of magnetorheological finishing pads (Figure 1b). Different from the rigid workpiece/abrasive plate contact in V-groove finishing, the contact between the ceramic ball and the semi-solid magnetorheological finishing pads was flexible. As a result, compared with conventional V-groove grinding, the surface finishing in different sections of the ball is evenly uniform because of the larger contact area between the magnetorheological finishing pads and the ball surface (Figure 1c).

### 2.2. Polishing Experiment

**Figure 2:**
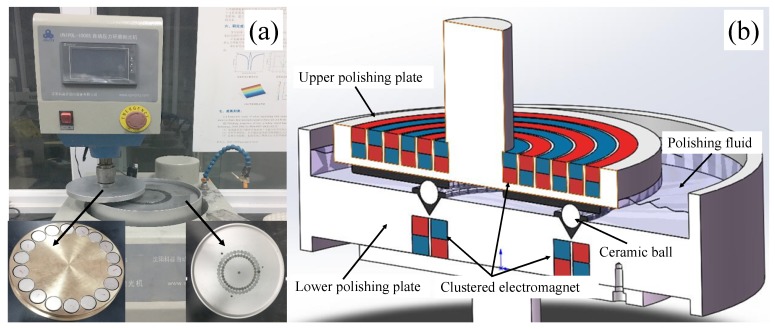
Experimental setup: (**a**) the machine tool of the clustered magnetorheological finish (**b**) cross-section of the CMRF machine.

A series of polishing experiments were conducted on a customized CNC CMRF machine (UNIPOL-1000S, Kejing, Shenyang, China). The machine tool was modified based on a V-groove finishing machine by assembling a series of circularly-distributed electromagnets was fixed on the two polishing plates, as shown in Figure 2a. The ceramic balls were placed in the V-ditch of the lower plate, and the magnetorheological finishing slurry, which consisted of abrasive grains (e.g., micro diamond particles), iron powder, and polishing fluid, was on the surface in the V-groove of the lower polishing plate (Figure 2b). During the polishing, the upper and lower polishing plates rotated around their spindles, respectively, and the spindle of the upper plate simultaneously revolved around the spindle of the lower plate. 

The workpiece used in this study was ${\mathrm{Si}}_{3}N_{4}$ balls fabricated by hot isostatic pressing (Shanghai Fanlian Technology Company, Ltd., Shanghai, China), with a diameter of 9.5 mm and surface roughness of 63 nm. The physical properties of the ${\mathrm{Si}}_{3}N_{4}$ ceramic balls obtained are listed in Table 1. Since the lack of information on the slurry component and quantities of CMRF in ${\mathrm{Si}}_{3}N_{4}$ ball polishing, The components and quantities of the slurry were selected based on the previous experimental results of our research group, which applied CMRF in polishing ceramic planes (SrTiO₃ and SiC) [16,17]. The magnetorheological slurry was made up with 400 mL polishing fluid and the mixed powders of 4% diamond abrasive grains (1 μm) and 16% hydroxyl iron powders (3 μm). The polishing fluid consisted of glycerol, water, alcohol and antirust agent (Fe_3_O_4_), and the volume fractions (%) of different components were listed in Table 2. To ensure the uniform dispersion of the abrasive grains and the iron particles in the slurry, the raw powders were mixed by ball milling for thirty minutes, and the magnetorheological liquid was processed by ultrasonic vibration (40 MHz) for another thirty minutes.

In the polishing of ceramic balls (e.g., V-groove polishing), the rotation speed of the upper and lower plates ($\omega_{A}$ and $\omega_{B}$), the gap between the upper and lower plates (*δ*) and the eccentricity of the upper and lower plates (*e*) are the main parameters which affect the quality of the surface finish, because these parameters affect the tool trace of the polishing. In this study, different $\omega_{A}$, $\omega_{B}$, *δ* and *e* were selected and adopted orthogonally to investigate their effects on the quality of final surface finish. As presented in Table 3, the range of each cutting parameter were determined according to the previous experiments [9,10], which were proved to be appropriate ranges for the polishing of ceramic balls. The rotation speeds of the upper and lower polishing plates ranged from 10 to 30 rpm and 20 to 60 rpm; the eccentricity and machining gap increased from 5 to 15 mm; machining gap ranged from 0.8 to 1.2 mm, all of which were optimized in our previous research. The intensity of magnetic field was controlled in-situ during the grinding process in order to ensure the stability of the magnetorheological pad during the CMRF process.

**Figure 3:**
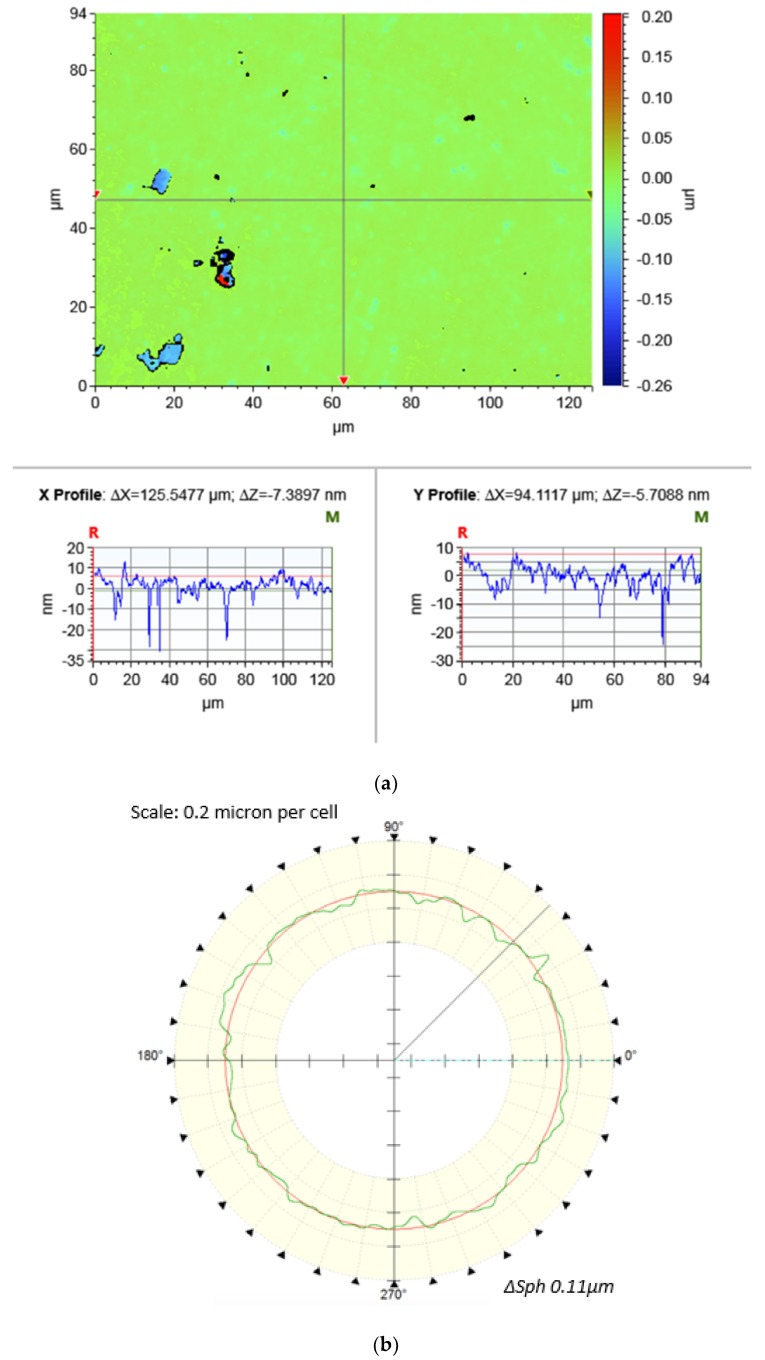
Results of measurement: (**a**) *Ra* measured by Contour GT-X3 (**b**) ∆*Sph* measured by Talyrond 585 LT.

The time of each polishing experiment was sixty minutes. After CMRF processes, the surface roughness and sphericity were measured, both of which were often used to evaluate the quality of the surface finishing of spherical parts in industry. The surface roughness (Ra) was measured with a a white light interferometer (Contour GT-X3, Bruker, Karlsruhe, Germany) and the sphericity (∆*Sph*) was measured by a roundness measuring instrument (Talyrond 585 LT, Taylor Hobson, West Chicago, IL, USA). Results of measurements of surface roughness and sphericity were presented in Figure 3, respectively. For each polished balls, the surface roughness was measured with ten randomly-selected positions and, thus, the reported Ra value was the average of ten measurements; the sphericity was determined by measuring the profiles of the great circle of the spherical surface, and the value was the average of ten-times measurement as well. 

### 2.3. Experimental Results and Discussion

Table 4 shows the results of surface roughness, sphericity and their standard errors. Processed by CMRF at different polishing parameters, the surface roughness reduced from 63 nm to the values between 4 and 13 nm, and sphericity values were between 0.11 and 0.18, both of which demonstrated that the CMRF could polish the ceramic balls with qualified surface finish (G5 Level, according to National Standard). Also, it was found that the lowest surface roughness (4.35 nm) and smallest spherical error (0.11 µm) was measured from the ball polished with rotation ratio 30 rpm/40 rpm, the eccentricity of 10 mm and machining gap of 0.8 mm.

Furthermore, significance analysis was conducted to evaluate the influence of different cutting parameters on surface roughness and sphericity. The factor of significance *R* was calculated by the following equation:

(1) $$R=\bar{K_{max}}-\bar{K_{min}}$$

where $\bar{K}$ was the average value of the measurement (*Ra* or sphericity ∆*Sph*) when one of the cutting parameters was fixed. For example, the average roughness at the machining gap 0.8 mm was calculated as: $\bar{K_{Ra}}\;10.67=\frac{8.663+10.63+12.70}{3}$.

From the results listed in Table 5, it could be found that the factor of significance of the four cutting parameters were 4.096 (*δ*), 2.661 (*e*), 2.57 ($\omega_{B}$), 1.772 ($\omega_{A}$), indicating that the influence of the machining gap on the surface roughness was more significant than other parameters. Similarly, the gap between the abrasive plates influenced more on the sphericity due to the largest significant factor (0.05) as well.

According to the significance analysis, it could be concluded that the finishing parameters employed in this study affected the surface roughness and sphericity in different ways. The machining gaps influenced the final surface finish by affecting the mechanical properties of magnetorheological pad. According to the findings of Pan et al. [21], the mechanical stiffness of the magnetorheological pad increased with the decrease of gap between the two magnetic poles because of the increase of the magnetic intensity. If the gap between the abrasive plates was small, the stiffness of the magnetorheological pad could be increased significantly. This could lead to a relative large friction force at the ball/pad interface, which consequently caused excessive scratches on machined surfaces. In contrast, the forces of abrasion became smaller when larger machining gaps were adopted, which caused the insufficient finishing of the spherical surface with the larger surface roughness (the *Ra* of Test 3 and Test 4). On the other hand, the eccentricity of the abrasive plates influenced the surface finish by changing the distribution of the magnetic field. It was reported in the study of Jain et al. [22] that the size of the magnetorheological bind was larger when the eccentricity of the magnetic poles became bigger. However, from the results of both simulation and experiment, it was observed that the difference between the surface finishing at the eccentricity of 10 mm and that of 5 mm was insignificant (Test 6 and Test 7). This suggested that the eccentricity of 10 mm was appropriate for the CMRF processes in this study. In addition, the rotating speeds governed the stability of the CMRF processes. With the application of larger rotating speed, the material removal rate could be increased because more abrasion occurred on the surface per unit of time. However, the magnetorheological pad is semi-solid, and the shape of the abrasive bind could be push away due to the centrifugal effect during rotation. This phenomenon could cause the reduction on the abrasive forces, which contributed to the higher surface roughness (the roughness of Test 3).

**Figure 4:**
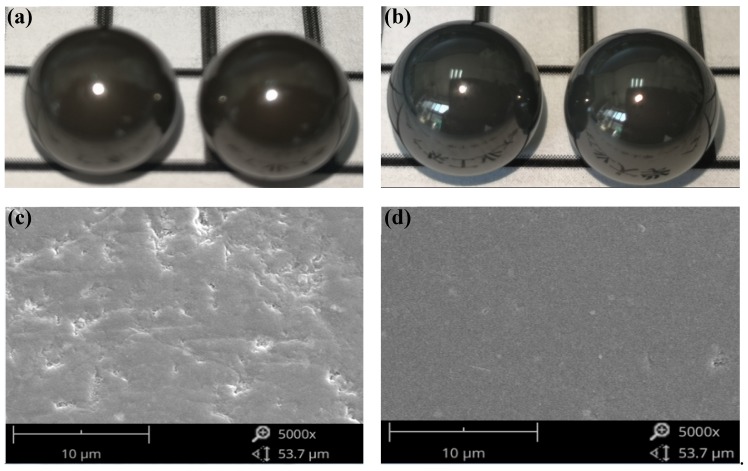
Optical images and micro morphologies of ceramic balls: (**a**) the ceramic balls before CMRF (**b**) the ceramic balls after CMRF (Test 2 and Test 7) (**c**) the micro morphology of unmachined surface (**d**) the micro morphology of polished surface (Test 6).

The optical images and micro morphologies of ceramic balls before and after the CMRF process are compared in Figure 3. It was observed that the polished surface of the ceramic balls exhibited a mirror-reflective effect after CMRF process (Figure 4b). Observations via scanning electron microscope analyses showed that dimples, craters, and cracks present on as-received spherical surface (Figure 4c) were almost completely removed by CMRF process with very few defects left (Figure 4d).

**Figure 5:**
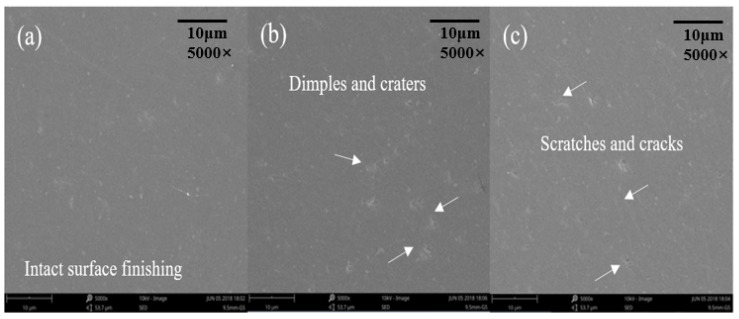
Miro morphology (5000 ×) of the polished surface (**a**) the surface of Test 9, (**b**) the surface of Test 6, (**c**) the surface of Test 3.

The finest surface finishing was found on the surface of No. 9 test (Figure 5a). Nonetheless, defects such as tiny dimples, fractures, and cracks could still be observed on the machined surfaces of the other eight tests. This could be attributed to the scratch and collision of the abrasive grains during the finishing processes. The material removal mechanism of ceramic materials in abrasive processes, in general, was fracture-dominated [23]. 

When polishing ${\mathrm{Si}}_{3}N_{4}$ ceramic balls, the abrasive grains could cause the crack genrations with the depth up to 15 µm at larger finishing parameters, resulting in the excessive fracture on the machined surface [24]. This means that those defects could be observed on surfaces polished with inappropriate selection of parameters. For example, obvious dimples were found when larger rotating speeds were adopted (Figure 5b); cracks and scratches were found at larger eccentricity of the abrasive plates (Figure 5c).

## 3. Modelling of CMRF Process

According to the experimental results, it could be suggested that the eccentricity, rotating speeds, and machining gap are three major parameters that affect the surface roughness and sphericity. To investigate the mechanism of the influence of different cutting parameters on the surface finishing, a mathematical model was developed to analyze the kinematic status of the balls at different eccentricities, rotating speeds, and machining gaps in the CMRF of spherical parts. 

### 3.1. Kinematics of the Ceramic Balls in CMRF Process

**Figure 6:**
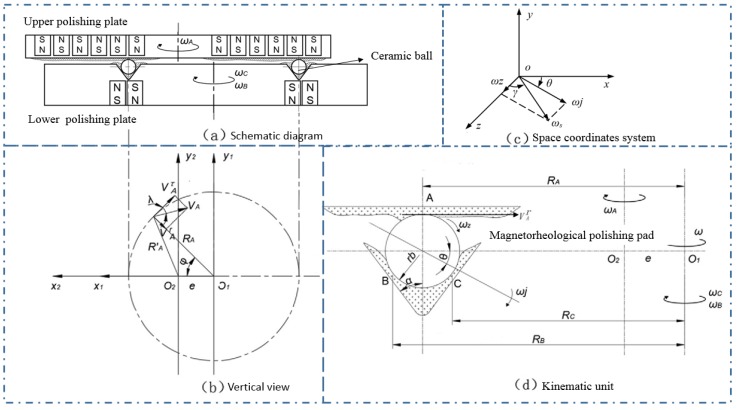
Schematic diagram of driving model of ceramic ball and finishing pad. (**a**) Schematic diagram. (**b**) Vertical view. (**c**) Space coordinates system. (**d**) Kinematic unit.

Figure 6 presents the kinematic status of a ceramic ball in the CMRF process. The upper plate and lower plate rotated around their own spindles with the angular speed $\omega_{A}$ and $\omega_{B}$ (Figure 6a). As shown in the top view of the polishing system (Figure 6b), *O_1_x_1_y_1_* and *O_2_x_2_y_2_* were two coordinates developed in the reference systems of upper plate and lower plate, respectively (*O_1_* and *O_2_* are the centers of the plates). In this study, the rotation status of three contact points one at the interface of the workpiece/upper magnetorheological finishing pad (point A) and other two at the interface of the workpiece/upper magnetorheological finishing pad (point B and point C) was modelled. The kinematics of the ceramic ball was investigated by analyzing the rotating speed *ω_z_* and *ω_j_*, which were the orthogonal components of and the rotating angle *θ* and *γ* (Figure 6c), and could significantly affect the enveloping development of the trace on spherical surface. 

The eccentricity *e* is defined as the distance between two points *O_1_* and *O_2_*. At a certain moment, the rotation angle of the ball billet around the V-shaped groove is *φ*, and the linear velocity *V_A_* of the point *A′* on the upper finishing plate is orthogonally decomposed, which were the tangential component of *V^τ^_A_*, and the radial component of *V^r^_A_*. The angle between *V_A_* and *V^τ^_A_* is denoted by *λ*; *R_A_* = *O_1_A*, *R′_A_* = *O_2_A*. Figure 6d illustrates the movement unit of the ball billet. The three contact points between the finishing pad and ball billet are marked with *A*, *B*, and *C*.

According to the assumption that no relative sliding at the contact point between the ball billets and finishing pad would occur, the kinematics equations can be written as follows:

(2) $$\{\begin{array}{c} V_{A}^{\tau }\omega_{A}=R_{A}\omega \omega_{A}+r_{b}\omega_{j}\omega_{B}\theta \\ R_{B}\omega_{B}=R_{B}\omega -r_{b}\omega_{j}\sin\left(\alpha +\theta \right)\\ R_{C}\omega_{C}=R_{C}\omega -r_{b}\omega_{j}\sin\left(\alpha -\theta \right)\\ V_{A}^{r}=\omega_{z}r_{b} \end{array}$$

where *R_A_*, *R_B_* and *R_C_* are the distances between the three contact points and the rotation axis of lower finishing plate are corresponded to *R_A_*, *R_B_*, and *R_C_*; *ω_A_*, *ω_B_* and *ω_C_* (*ω_B_* = *ω_C_*) are the angular speed of A, B and C; the and these rotation speeds are corresponded to *ω_A_*, *ω_B_*, and *ω_C_* (*ω_B_* = *ω_C_*), respectively, The ball billet with a radius *r_b_* self-rotated at an angular velocity *ω_s_*, and the shape of the V-groove is determined by the groove half-angle *α*. *V^r^_A_ = V_A_cosλ, V^r^_A_ = V_A_sinλ, V_A_ = R’_A_ω_A_:*

(3) $$\{\begin{array}{l} V_{A}^{\tau }=V_{A}cos\lambda \\ V_{A}^{r}=V_{A}sin\lambda \\ V_{A}=R_{A}^{\prime }\omega_{A} \end{array}$$

and there are the following geometric relations in Δ*O_1_O_2_A*:

(4) $$\{\begin{array}{c} R_{A}^{\prime }\cos\lambda =V_{A}-e\cos\Phi \\ R_{A}^{\prime }\sin=e\sin\Phi \end{array}$$

(5) $$\{\begin{array}{l} R_{B}=R_{A}+r_{b}\cos\alpha \\ R_{C}=R_{A}-r_{b}\cos\alpha \end{array}$$

Thist can be solved by Equation (2) to Equation (5):

(6) $$\left\{ \begin{array}{l} \theta =\tan^{-1}\left\{\frac{\begin{array}{c} \left[R_{A}R_{B}\omega_{B}-\left(R_{A}-e\cos\Phi \right)R_{B}\omega_{B}\right]\sin\alpha -\left[R_{A}R_{C}\omega_{C}-\left(R_{A}-e\cos\Phi \right)R_{C}\omega_{A}\right]\sin\alpha \\ -R_{A}R_{B}\left(\omega_{C}-\omega_{B}\right) \end{array}}{\left[R_{A}R_{B}\omega_{B}-\left(R_{A}-e\cos\Phi \right)R_{B}\omega_{B}\right]\cos\alpha -\left[R_{A}R_{C}\omega_{C}-\left(R_{A}-e\cos\Phi \right)R_{C}\omega_{A}\right]\cos\alpha }\right\}\\ \gamma =\tan^{-1}\left(\frac{\omega_{j}}{\omega_{Z}}\right)=\tan^{-1}\left\{\frac{R_{A}\left(R_{B}\omega_{B}+R_{C}\omega_{C}\right)-\left(R_{A}-e\cos\Phi \right)\left(R_{B}+R_{C}\right)\omega_{A}}{e\omega_{A}\sin\Phi \left[\left(R_{B}+R_{C}\right)\cos\theta +R_{A}\sin\left(\alpha +\theta \right)+R_{A}\sin\left(\alpha -\theta \right)\right]}\right\} \end{array}\right.$$

and the rotating speeds $\omega_{j}$ and $\omega_{Z}$ were expressed with the following equations:

(7) $$\{\begin{array}{c} \omega_{j}=\frac{\left(R_{B}\omega_{B}+R_{C}\omega_{C}\right)-2\left(R_{A}-e\cos\Phi \right)\omega_{A}}{2r_{b}\left(1+\sin\alpha \right)\cos\theta }\\ \omega_{Z}=\frac{e\omega_{A}\sin\Phi }{r_{b}} \end{array}$$

Based on Equations (6) and (7), it was found that when the groove half-angle *α* is fixed, the motion of sphere could be controlled by changing the position radius of the groove *R_A_*, the eccentricity *e* between the two rotating plates, and the rotation speeds *ω_A_* and *ω_B_* (or *ω_C_*). Acordingly, a high efficiency and uniform spherical envelope model could be achieved. In addition, the magnetic field intensity, magnetorheological finishing force, and machining gap *δ* can be adjusted, then the surface roughness and spherical error of the silicon nitride ceramic balls could be optimized and controlled.

### 3.2. Kinematic Simulation in ADAMS

**Figure 7:**
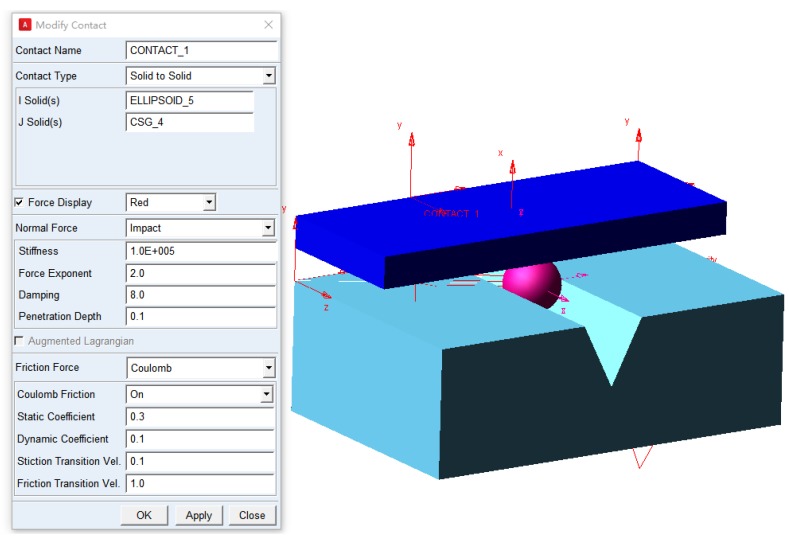
Modelling of CMRF process in ADAMS.

The movement of the three contact points were simulated by using the software ADAMS (MSC Software Corporation, Newport Beach, CA, USA) to present the visualized kinematic status of the ${\mathrm{Si}}_{3}N_{4}$ ceramic ball. As presented in Figure 4, the three-dimensional solid models of the ball and two magnetorheological pads designed in Solidworks (SolidWorks Inc., Waltham, MA, USA) were imported into ADAMS. The contact forces directly affect the accuracy of the simulation of the interaction between ball billet and finishing pad. The equivalent contact forces at these three points were calculated by using the impact function of ADAMS. The contact force parameters include the stiffness coefficient, force exponent, damping, penetration depth, static/dynamic coefficient, and stiction/friction transition velocity, as shown in Figure 7.

The material properties of ${\mathrm{Si}}_{3}N_{4}$ (Table 1) and the magnetorheological pads (Table 6) were pre-defined. Thus, the software ADAMS could automatically calculate the mass and moment inertia of the components. Based on the contact collision model [25,26], when the contact occurs between two entities, the ADAMS will also automatically calculate the geometric center of contact intersection. The polishing parameters were pre-defined with the values listed in Table 7. According to above established kinematic model, different finishing parameters such as the eccentricity between the two abrasive plates, rotation speed ratio *ω_A_*/*ω_B_*, and machining gap were selected to simulate and optimize the finishing trajectory by using single variable method.

Based on above spherical envelope model and the contact force parameters, the trace of three contact points (*A*, *B*, and *C*) between the ball billet and the magnetorheological finishing pad can be simulated by ADAMS. The simulation time is set as 30 s with a step size of 0.01, and the function expression of finishing speed is listed in Table 8.

### 3.3. Formation of the Trace and Distribution of the Contact Points

The uniform abrasion of the spherical surface is important to the quality of the surface finishing of ${\mathrm{Si}}_{3}N_{4}$ balls. As a result, it is important to ensure the even distribution of the finishing traces on different areas of the spherical sections [27,28]. In this study, the simulation results were evaluated by analyzing the visualized trace and the distribution of the contact point.

**Figure 8:**
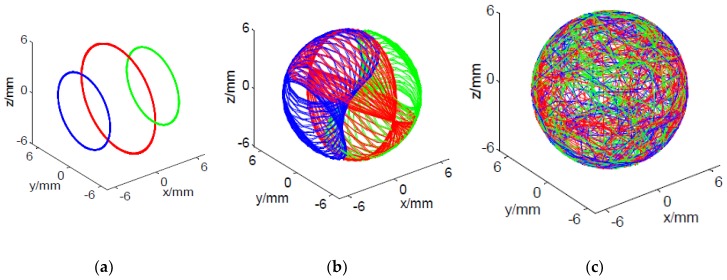
Traces of Contact points in different finishing methods (**a**) centric V-groove finish (**b**) eccentric V-groove finish (**c**) CMRF.

Figure 8 shows the visualized tool trace of three finishing methods. Result shows that it is impossible to realize the uniform finishing of the spherical surface via conventional V-groove finishing method. This is because the traces of the contact points are three concentric circles due to the rigid three-point contact between the ceramic ball surface and the abrasive plate, which cannot envelop the spherical surface (Figure 8a). In contrast, relatively large areas of the spherical surface were enveloped by the tool trace of eccentric V-groove finish (Figure 8b). This is because the eccentricity of the two abrasive plates increased the range of the rotating angels *θ* and *φ*, which subsequently increased the area of envelopment by the tool trace [29,30]. As for CMRF method, the range of the rotating angel is larger than that of eccentric V-groove finish due to the combination of the plates eccentricity and the flexible of the contact between the ball surface and magnetorheological pads, which is in turn readily to form a fully-enveloped tool trace on the spherical surface [31]. In this study, the visualization results of the tool trace of CMRF were generated and analyzed (Figure 8c). The positions of contact points at different times were exported from the ADAMS. Then, the trace of the point A, point B and point C could be plotted in MATLAB, and the entire finishing trace was synthesized with the traces of the three contact points.

**Figure 9:**
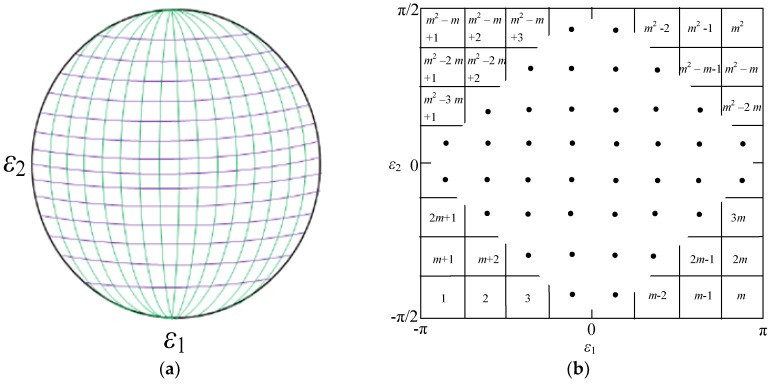
(**a**) the longitude-latitude meshing on a sphere (**b**) the *m × m* meshing of the spherical surface.

Besides the visualized results of kinematic simulation, the contact points in different regions of the surface were counted to evaluate the uniformity of the finishing, which influenced the surface roughness in different areas and the overall sphericity. As shown in Figure 9, the spherical machined surface was divided into m × m sections with the same size by the longitude-latitude meshing method [32]. The surface was divided into 16 sections in both longitude direction ($\varepsilon_{1}\in \left[-\pi ,\;\;\pi \right]$) and latitude direction ($\varepsilon_{2}\in \left[-\frac{\pi }{2},\;\;\frac{\pi }{2}\right]$), and the area of the meshed unit could be expressed with the following equation:

(8) $$A_{ij}=\int_{\varepsilon_{1}^{i}}^{\varepsilon_{1}^{i+1}}\int_{\varepsilon_{2}^{i}}^{\varepsilon_{2}^{i+1}}r_{b}^{2}\cos\varepsilon_{2}d\varepsilon_{1}d\varepsilon_{2}=\frac{2\pi }{m}r_{b}^{2}\left|\sin\varepsilon_{2}\left(j+1\right)-\sin\varepsilon_{2}\left(j\right)\right|$$

The positions of the contact points at the time $t_{i}$ was calculated with the following equation:

(9) $$P_{i}=P_{0}M\left(f_{1},\;\varphi_{1}\right)M\left(f_{2},\;\varphi_{2}\right)\cdots M\left(f_{i-1},\;\varphi_{i-1}\right)$$

where $P_{i}$ was the position of the contact point on the spherical surface at $t_{i}$, $P_{0}$ was the initial position of the contact point and $M\left(f_{n},\;\varphi_{n}\right)$ was the rotation transforming function, which could be determined by the rotating vector $f_{n}$ and the rotating angel $\varphi_{n}$ (*n* = 1, 2, 3 …). Both $f_{n}$ and $\varphi_{n}$ could be exported from the results of ADAMS, and the positions of the contact points A, B and C at different time could be then calculated. Furthermore, the standard deviation of the number of contact points in each region was obtained through numerical calculation with the following equation:

(10) $$SD=\sqrt{\frac{\sum {\left(Q_{i}-\bar{Q}\right)}^{2}}{n-1}},\;i=1,2,3\ldots ...n$$

where $\bar{Q}=\sum Q_{i}/n$ is the average value of points in each region divided on the sphere. The smaller the value of *S_Q_*, the better the finishing uniformity of the whole sphere.

### 3.4. The Visualized Traces and Distributions of the Contact Points

**Figure 10:**
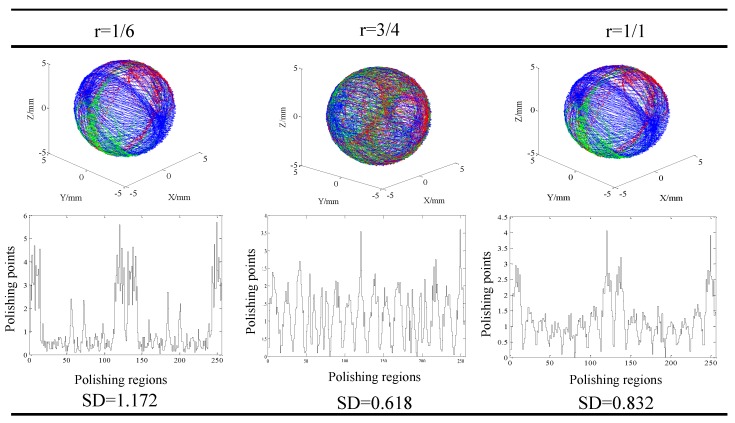
Distribution of the finishing traces at different rotating ratios when eccentricity and machining gap were fixed (*e* = 10 mm, *δ* = 0.8 mm).

**Figure 11:**
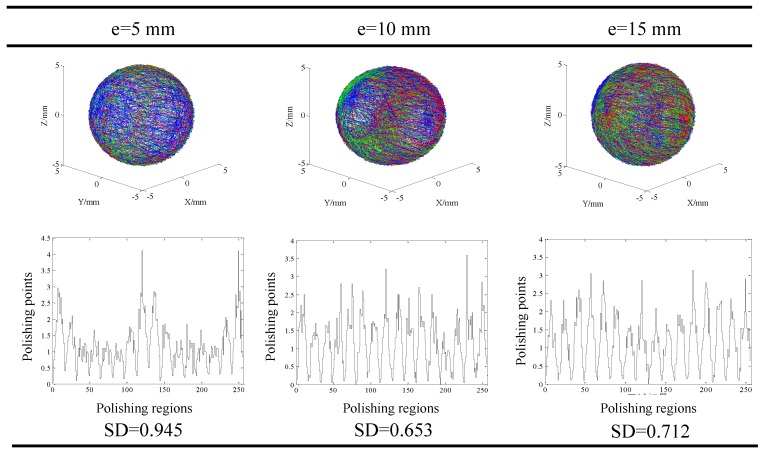
Distribution of the finishing traces at different eccentricities when the rotating ratio and machining gap were fixed (*δ* = 0.8 mm, *r* = 3/4).

**Figure 12:**
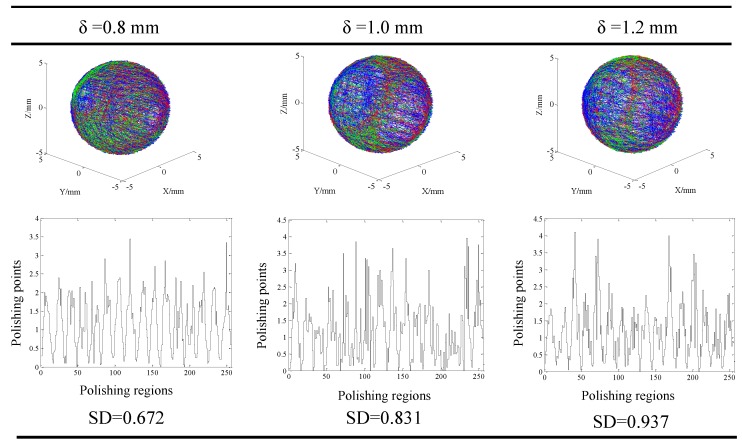
Distribution of the finishing traces at different gaps when rotating ratio and eccentricity were fixed (*e* = 10 mm, *r* = 3/4).

Result of the visualized trace of the contact points and distribution of the contact points in different regions at different machining parameters are shown in Figure 10, Figure 11 and Figure 12. Generally, it could be found that the traces of the contact points at different cutting parameters enveloped the spherical surface entirely, which suggested that the CMRF method ensured the sphericity of ceramic balls after surface finishing process. However, the numbers of the contact points in different regions were significantly affected by the adoption of the polishing parameters. When different ratios of the rotating speeds were adopted, it was obvious that contact points were uniformly distributed at rotation of ¾. In contrast, the numbers of contact points were larger in the regions No. 1 to No. 25, No. 110 to No. 140 and No. 230 to No. 256 at the rotation ratios of 1/6 and 1/1. This was in accordance with the visualized results that the traces of point B (green) and Point C (red) distributed intensively in limited regions (Figure 10). As for the effect of different eccentricities, it could be seen that the contact points were uniformly-distributed when larger values, 10 mm and 15 mm, were used (Figure 11). The distribution of the contact points showed different trend when different machining gaps were adopted. The contact points fluctuated obviously when the smallest machining gap was adopted (Figure 12). According to the calculation of *SD*s, the simulative results could be compared further. Specifically, the machining gap exhibited the most significant influence on the distribution of the contact points, which was reflected by the 0.5 on the variance of *SD*s ($\Delta SD=SD_{largest}-SD_{smallest}$).

This is in accordance with the analysis of significance. The variance of ΔSD was relative small when using different eccentricities and machining gaps, both of which were around 0.25. As a result, it could be concluded that the best surface finishing could be obtained with the adoption of the polishing parameters of rotation ratio of ¾, eccentricity of 10 mm, and machining gap of 0.8 mm due to the smallest *SD*. This was in accordance with the experimental results that the roughness and sphericity were the smallest when polishing the balls with the parameters set.

## 4. Conclusions

The newly developed polishing method, clustered magnetorheological finishing (CMRF), was applied to polish ${\mathrm{Si}}_{3}N_{4}$ ceramic balls in this study. The surface finishing of ceramic balls using different finishing parameters was investigated experimentally and theoretically. The ${\mathrm{Si}}_{3}N_{4}$ balls were polished using the CMRF method with orthogonal polishing parameters, and the surface finishing conditions including roughness, sphericity and microstructure morphology were examined. An analytical model describing the kinematics of the contact points were developed, and the visualized traces of the contact points and the distribution of the points in different regions were obtained. The findings was concluded as follows:

(1) It was found that the surface roughness of ${\mathrm{Si}}_{3}N_{4}$ balls was reduced from 63 nm to 4.35 nm and the sphericity was reduced from 0.18 μm to 0.11 μm when the parameters of rotation speed 30 rpm/40 rpm, eccentricity of 10 mm and polishing gap of 0.8 mm were applied.
(2) The finishing parameters affected the surface roughness and sphericity. The adoption of larger machining gaps increased the surface roughness due to the decrease of the stiffness of the magnetorheological pads. The eccentricity influenced the surface finish by changing the distribution of the magnetic field, and the ratio of the rotating speeds influenced the stability of the CMRF processes.
(3) The modelling and simulation of CMRF showed the visualized traces and distributions of the contact points with different polishing parameters. The distribution of contact points in different sections showed that the contact points were uniformly distributed when the machining gap, rotation speed and eccentricity of the polishing plates were correctly selected. The best simulation results were found using the same parameter set as in the experimental results.

## Figures and Tables

**Table 1:** The physical properties of ${\mathrm{Si}}_{3}N_{4}$ [20].

Density	Hardness	Elastic Modulus	Toughness	Flexure Strength
1.8–3.22 g/cm^3^	18 Gpa	284–460 Gpa	6–10.5 MPa·m^1/2^	550 MPa

**Table 2:** Fractions of different components in finishing solution [16,17].

Material	Water	Glycerol	Antirust Agent	Dispersant
Fraction	80%	10%	5%	5%

**Table 3:** Machining parameters of the orthogonal finishing experiments.

Test No.	Machining Gap (mm)	Rotation Speed of Upper Plate (r/min)	Rotation Speed of Lower Plate (r/min)	Eccentricity (mm)
1	1.2	10	60	5
2	1.2	20	40	10
3	1.2	30	20	15
4	1.0	10	40	15
5	1.0	20	20	5
6	1.0	30	60	10
7	0.8	10	20	5
8	0.8	20	60	15
9	0.8	30	40	10

**Table 4:** Surface roughness and sphericity of the balls after finishing.

Test No.	Roughness *Ra* (nm)	Standard Deviation of *Ra* (nm)	Sphericity ∆*Sph* (μm)	Standard Deviation of ∆*Sph* (μm)
1	8.683	1.233	0.18	0.02
2	10.63	0.811	0.13	0.01
3	12.70	1.864	0.16	0.02
4	13.44	0.538	0.15	0.01
5	11.46	0.716	0.18	0.02
6	11.17	0.297	0.13	0.02
7	11.42	0.554	0.15	0.03
8	8.004	1.032	0.12	0.02
9	4.350	0.909	0.11	0.01

**Table 5:** Analysis of the significance of the cutting parameters.

Machining Parameters		Machining Gap	Rotation Speed of Upper Plate	Rotation Speed of Lower Plate	Eccentricity
Average of *Ra* (nm)	$\bar{K_{Ra}}$_1_	10.67	11.179	9.287	10.519
	$\bar{K_{Ra}}$_2_	12.02	10.03	9.472	8.718
	$\bar{K_{Ra}}$_3_	7.924	9.407	11.86	11.379
Significance	*R_Ra_*	4.096	1.772	2.57	2.661
Average of ∆*Sph* (μm)	$\bar{K_{∆\mathrm{Sph}}}$	0.16	0.16	0.14	0.17
	$\bar{K_{∆\mathrm{Sph}}}$	0.15	0.15	0.13	0.12
	$\bar{K_{∆\mathrm{Sph}}}$	0.13	0.13	0.16	0.15
Significance	*R*_∆*Sph*_	0.03	0.03	0.03	0.05

**Table 6:** The physical properties of magnetorheological pads [23].

Density	Stiff-ness	Force Exponent	Damping	Static Coefficient	Dynamic Coefficient	Stiction Transition	Friction Transition
7.845 g/cm³	$10^{-5}$	2.0	8.0	0.3	0.1	0.1	1.0

**Table 7:** Finishing parameters in the simulation.

Test No.	Ratio of Rotating Speed	Eccentricity (*e*)/mm	Machining Gap (*δ*)/mm
1	1/6	10	0.8
2	3/4	10	0.8
3	1/1	10	0.8
4	3/4	10	1.0
5	3/4	10	1.2
6	3/4	5	0.8
7	3/4	15	0.8

**Table 8:** Function expression of finishing speed.

Finishing Speed		Function Expression
Upper plate	*ω_A_*	(step(time, 0, 0, 5, π*ω_A_*/30)+step(time, 5, 0, 100, 0)) × time
Lower plate	*ω_B_*	(step(time, 0, 0, 5, π*ω_B_*/30)+step(time, 5, 0, 100, 0)) × time

## References

[1] Tian C., Liu N., Lu M. (2008). Effect of WC on microstructure and mechanical properties of silicon nitride nano-composites. J. Mater. Process. Technol..

[2] Tapasztó O., Tapaszto L., Lemmel H., Puchy V., Dusza J., Balázsi C., Balázsi K. (2016). High orientation degree of graphene nanoplatelets in silicon nitride composites prepared by spark plasma sintering. Ceram. Int..

[3] Zhang B., Nakajima A. (2003). Dynamics of magnetic fluid support grinding of Si3N4 ceramic balls for ultraprecision bearings and its importance in spherical surface generation. Precis. Eng..

[4] Yuan J., Lü B., Lin X., Zhang L., Ji S. (2002). Research on abrasives in the chemical–mechanical polishing process for silicon nitride balls. J. Mater. Process. Technol..

[5] Zhuo Y., Zhou X., Yang C. (2014). Dynamic analysis of double-row self-aligning ball bearings due to applied loads, internal clearance, surface waviness and number of balls. J. Sound Vib..

[6] Levesque G., Arakere N.K. (2008). An investigation of partial cone cracks in silicon nitride balls. Int. J. Solids Struct..

[7] Oh D.-S., Kang K.-H., Kim H.-J., Kim J.-K., Won M.-S., Kim D.-E. (2018). Tribological characteristics of micro-ball bearing with V-shaped grooves coated with ultra-thin protective layers. Tribol. Int..

[8] Feng K.P., Zhou Z.Z., Lv B.H., Yuan J.L. (2013). Study on Dual-Plane Ball Polishing Method for Finishing Ceramics Ball. Adv. Mater. Res..

[9] Zhao P., Guo W., Feng M., Lv B., Deng Q., Yuan J. (2013). A Novel Lapping Method for High Precision Balls Based on Variable-Radius V-Groove. J. Micro Nano-Manuf..

[10] Feng M., Wu Y., Yuan J., Ping Z. (2017). Processing of high-precision ceramic balls with a spiral V-groove plate. Front. Mech. Eng..

[11] Guo H., Wu Y., Lu N., Fujimoto M., Nomura M. (2014). Effects of pressure and shear stress on material removal rate in ultra-fine polishing of optical glass with magnetic compound fluid slurry. J. Mater. Process. Technol..

[12] Miao C., Lambropoulos J., Jacobs S.D. (2010). Process parameter effects on material removal in magnetorheological finishing of borosilicate glass. Appl. Opt..

[13] Ranjan P., Balasubramaniam R., Jain V. (2017). Analysis of magnetorheological fluid behavior in chemo-mechanical magnetorheological finishing (CMMRF) process. Precis. Eng..

[14] Umehara N., Kirtane T., Gerlick R., Jain V., Komanduri R. (2006). A new apparatus for finishing large size/large batch silicon nitride (Si3N4) balls for hybrid bearing applications by magnetic float polishing (MFP). Int. J. Mach. Tools Manuf..

[15] Kordonski W.I., Gorodkin S. (2011). Material removal in magnetorheological finishing of optics. Appl. Opt..

[16] Pan J., Yu P., Yan Q., Li W. (2017). An experimental analysis of strontium titanate ceramic substrates polished by magnetorheological finishing with dynamic magnetic fields formed by rotating magnetic poles. Smart Mater. Struct..

[17] Liang H., Lu J., Pan J., Yan Q. (2017). Material removal process of single-crystal SiC in chemical-magnetorheological compound finishing. Int. J. Adv. Manuf. Technol..

[18] Zhao F., Zhou L., Fan Z., Dai Z. (2018). Research on Surface Processing of Quartz Wafer Based on Magnetorheological Finishing and Ion Beam Figuring. Procedia CIRP.

[19] Wang T., Cheng H., Dong Z.-C., Tam H.Y. (2013). Removal character of vertical jet polishing with eccentric rotation motion using magnetorheological fluid. J. Mater. Process. Technol..

[20] Zhang X., Wen D., Shi Z., Li S., Kang Z., Jiang J., Zhang Z. (2020). Grinding performance improvement of laser micro-structured silicon nitride ceramics by laser macro-structured diamond wheels. Ceram. Int..

[21] Pan J., Yan Q. (2015). Material removal mechanism of cluster magnetorheological effect in plane polishing. Int. J. Adv. Manuf. Technol..

[22] Jain V., Ranjan P., Suri V., Komanduri R. (2010). Chemo-mechanical magneto-rheological finishing (CMMRF) of silicon for microelectronics applications. CIRP Ann..

[23] Li G., Li N., Wen C., Ding S. (2017). Investigation and modeling of flank wear process of different PCD tools in cutting titanium alloy Ti6Al4V. Int. J. Adv. Manuf. Technol..

[24] Kumar A., Ghosh S., Aravindan S. (2019). Experimental investigations on surface grinding of silicon nitride subjected to mono and hybrid nanofluids. Ceram. Int..

[25] Chen Y., Li J., Chen J., Xu L. (2018). Improving the Electrical Contact Performance for Amorphous Wire Magnetic Sensor by Employing MEMS Process. Micromachines.

[26] Flores P., Ambrósio J. (2010). On the contact detection for contact-impact analysis in multibody systems. Multibody Syst. Dyn..

[27] Muthunilavan N., Rajaram G. (2017). Effect on lubrication regimes with silicon nitride and bearing steel balls. Tribol. Int..

[28] Konstantinou G., Milenko K., Kosma K., Pissadakis S. (2018). Multiple Light Coupling and Routing via a Microspherical Resonator Integrated in a T-Shaped Optical Fiber Configuration System. Micromachines.

[29] Lee R.-T., Hwang Y.-C., Chiou Y.-C. (2006). Lapping of ultra-precision ball surfaces. Part I. Concentric V-groove lapping system. Int. J. Mach. Tools Manuf..

[30] Fu Q., Guo S., Zhang S., Hirata H., Ishihara H. (2015). Characteristic Evaluation of a Shrouded Propeller Mechanism for a Magnetic Actuated Microrobot. Micromachines.

[31] Lee R.-T., Hwang Y.-C., Chiou Y.-C. (2006). Lapping of ultra-precision ball surfaces. Part II. Eccentric V-groove lapping system. Int. J. Mach. Tools Manuf..

[32] Kritsikis E., Aechtner M., Meurdesoif Y., Dubos T. (2017). Conservative interpolation between general spherical meshes. Geosci. Model Dev..

